# The Effectiveness of an After-school Sport Sampling Intervention on Urban Middle School Youth in the Midwest: Posttest-Only Study

**DOI:** 10.2196/42265

**Published:** 2023-01-25

**Authors:** Joseph Lightner, Katlyn Eighmy, Ella Valleroy, Bridget Wray, Amanda Grimes

**Affiliations:** 1 School of Nursing and Health Studies University of Missouri-Kansas City Kansas City, MO United States; 2 Department of Population Health University of Kansas Medical Center Kansas City, KS United States

**Keywords:** physical activity, adolescent, sport sampling, physical literacy, BMI, health intervention, parenting, healthy lifestyle, youth, health inequality, underserved population

## Abstract

**Background:**

Effective and scalable interventions are needed to combat chronic low levels of youth physical activity. After-school sport sampling programs may be vital interventions for teaching sports and increasing physical literacy and physical activity, which result in healthy lifelong habits that are maintained into adulthood.

**Objective:**

The purpose of this study was to test the effectiveness of an after-school sport sampling intervention among underserved youth in the Midwest.

**Methods:**

Youth (n=81) in 3 middle schools within a large Midwest city participated in an 8-month, after-school physical activity intervention that aimed to increase moderate- and vigorous-intensity physical activity, improve physical literacy, and decrease BMI. Difference scores for this 2-group, posttest-only design were calculated. A series of 2-tailed *t* tests were conducted to assess between-group differences.

**Results:**

The intervention group had significantly better physical literacy (*t*_115_=7.57; *P*=.004) and engaged in more moderate- and vigorous-intensity physical activity minutes per week (*t*_115_=4.28; *P*=.04) and steps per day (*t*_115_=4.29; *P*=.03).

**Conclusions:**

An after-school sport sampling program may be an effective solution for combating youth physical inactivity. Future research should assess the scalability of this intervention with larger populations and in different areas.

**International Registered Report Identifier (IRRID):**

RR2-10.2196/37126

## Introduction

Nationally, 71.3% of middle schoolers do not meet physical activity recommendations [1]. Low levels of physical activity have contributed to increased rates of poor health, chronic disease, and obesity [2]. Obesity rates among youth have risen by nearly 7% in the last 20 years [3]. In 2018, over 1 in 5 youth (21.2%) were obese. Racial and ethnic minority youth are disproportionately affected by weight status. In 2017, it was reported that 37.7% of Black youth and 38.8% of Hispanic youth were overweight or obese, whereas 27.7% of White youth were overweight or obese [1]. Similarly, concerning trends are seen in physical activity rates. Physical activity rates have decreased by 5.5% among adolescents in the last 8 years alone, with only 21.1% of Black youth, 20.9% of Hispanic youth, and 25.6% of White youth engaging in at least 1 hour of moderate to vigorous physical activity (MVPA) per day in 2019 [4].

Evidence suggests that sports participation is associated with increased physical activity [5,6]. Therefore, playing sports outside of school hours is a promising strategy for physical activity interventions. However, sports participation differs by race, ethnicity, gender, and socioeconomic status [7,8]. Racial and ethnic minority students, as well as those who are socioeconomically disadvantaged, have lower rates of participation in sports [7]. Adolescent girls also participate less in sports teams than adolescent boys [8]. To increase participation, middle schoolers recommend that programming should incorporate a variety of sports in noncompetitive environments [9].

A barrier to sports participation is lacking the skills to be able to have fun and enjoy playing sports [10]. The “motivation, confidence, physical competence, knowledge, and understanding to value and take responsibility for engagement in physical activities for life” are determinants of physical literacy [11]. Physical literacy is increasingly recognized as a core construct in physical activity interventions and sports programming [12-14]. Overall, children have low levels of physical literacy, with 85% demonstrating inadequate levels [15]. Little is known about the relationship between physical literacy and long-term health outcomes, but emerging evidence suggests that physical literacy is related to physical activity levels and other health outcomes [16]. Although there has been a robust scientific conversation around the concept of physical literacy, more research is needed to explore relationships among physical literacy, physical activity, and health outcomes [17].

Sport sampling interventions may be a potential mechanism for increasing physical activity and physical literacy among youth. However, to date, a school-based, culturally tailored, participant-informed sport sampling intervention has not been evaluated for effectiveness. Therefore, the purpose of this study was to examine the effectiveness of an after-school, culturally tailored, participant-informed sport sampling program on physical activity, physical literacy, and BMI among underserved racial and ethnic minority youth in the Midwest.

## Methods

### Study Design, Settings, and Participants

The *Move More, Get More* study was conducted as a 2-group, posttest-only study. A total of 3 middle schools with grades 6 to 8 in the Kansas City Public School District (KCPS) participated in this study. All participating schools were public, but one was classified as a KCPS signature school that focuses on college preparation. All students at the three schools were eligible to participate in the intervention. Schools were recruited through the school district and identified as schools in the most need of programming to increase physical activity. The KCPS is the 12th largest school district in Missouri and primarily serves ethnic minority, low-income, and inner-city youth [18]. Over 36 languages are spoken in the schools, with 22% of students receiving English language learner services. All students qualify for free lunches or lunches at reduced costs [18]. Due to the nature of this study, we were unable to collect baseline physical activity data because students began the intervention on the day that they were provided with accelerometers. Therefore, we used end point assessments to compare the intervention group to the control group.

### Ethics Approval

All procedures of this study were approved by the University of Missouri-Kansas City Institutional Review Board (protocol number: 2017528).

### Intervention

The intervention aimed to increase the overall physical activity levels of middle school youth. Each school had a minimum of 2 trained sport instructors for leading after-school sport sampling sessions. Youth were not separated by gender, and youth only attended activities at their school. Table 1 shows the activities provided. The activities included equipment-based sports (basketball, soccer, football, etc), dance, yoga, and team-based games. The activities were selected and adapted based on student interest, culture, available facilities, instructor expertise, and available equipment. Sessions were held in a variety of settings at the school, including outdoor fields, indoor gymnasiums, hallways, and classrooms. Session locations varied depending on weather conditions and availability, which was based on other after-school programming.

The dose of the intervention was adapted for each school based on the schools’ release times and bus schedules. The first school hosted two 1-hour sessions each week. The second school hosted three 1-hour sessions each week. The third school hosted three 2-hour sessions each week. After-school sessions were held from September 2021 through May 2022 and aligned with the KCPS academic calendar. No COVID-19 restrictions (eg, social distancing), other than masking, were imposed by schools or public health agencies. Masking requirements ended in March 2022.

**Table 1:** Activities by school.

Study week	School 1 activities	School 2 activities	School 3 activities
1	Basketball	Basketball	Basketball
2	Basketball	Basketball and flag football	Basketball
3	Flag football	Basketball and flag football	Flag football
4	Flag football	Basketball	Flag football
5	Soccer	Dodgeball and basketball	Softball and baseball
6	Soccer	Basketball	Basketball
7	Capture the flag	Kickball	Yoga and aerobics
8	Games (red light, green light; shark and minnows; and tag)	Basketball	Yoga and aerobics
9	Games (red light, green light; shark and minnows; and tag)	Basketball	Frisbee, jump rope, and dance
10	Dodgeball and soccer	Flag football and kickball	Frisbee, jump rope, and dance
11	Games (red light, green light; shark and minnows; and tag)	Lacrosse and kickball	Soccer, frisbee, and dance
12	Mini-competition of basketball, soccer, football, and frisbee	Lacrosse and kickball	Soccer, frisbee, and dance
13	Mini-competition of basketball, soccer, football, and frisbee	Yoga, jump rope, and dodgeball	Free play
14	Canceled by school	Canceled by school	Canceled by school
15	Jump rope	Indoor games and trashball	Canceled by school
16	Jump rope and jump rope games	Indoor games and trashball	Aerobics, dance, and yoga
17	Basketball	Soccer	Aerobics, dance, and yoga
18	Basketball	Soccer	Jump rope and jump rope games
19	Flag football and basketball	Basketball	Kickball and basketball
20	Flag football	Basketball	Relay races
21	Basketball	Flag football	Basketball
22	Basketball and kickball	Flag football	Basketball
23	Circuit training and basketball	Badminton	Soccer and handball
24	Soccer	Volleyball	Soccer and handball
25	Soccer	Volleyball	Olympics week
26	Kickball	Kickball	Olympics week
27	Kickball	Kickball	Football
28	Ultimate frisbee	Ultimate frisbee	Football
29	Ultimate frisbee or dodgeball	Ultimate frisbee	Dodgeball and volleyball
30	Free play or review of sports	Free play or review of sports	Dodgeball and volleyball
31	Free play or review of sports	Free play or review of sports	Free play or review of sports

### Recruitment and Enrollment

Participants were recruited in August and September 2021 during multiple school events, such as the district enrollment fair, school lunches, and parent-teacher conferences. Parents provided written consent for students to participate in this study. Students also provided written and verbal assent before participating in this study. Participants completed a web-based questionnaire and an objective physical literacy assessment at baseline.

### Incentives

Participants received a US $25 gift card upon completion of their enrollment and follow-up data collection. A Garmin Vivofit 4 (Garmin Ltd) was provided as an incentive at baseline. Participants were also entered into a raffle giveaway for a US $150 gift card.

### Measures

#### Physical Activity

Physical activity was assessed with Garmin Vivofit 4 accelerometers throughout the study period. Daily steps and active minutes data were aggregated at the week level. To account for nonwear days, a daily mean was calculated based on a given week. Detailed accelerometer procedures can be found in the previously published protocol [19]. Similar accelerometer procedures were used in previous studies [20].

#### Physical Literacy

Physical literacy was assessed objectively by trained research assistants using the PLAYbasic instrument [21]. PLAYbasic assesses the physical abilities of participants in the following four domains: locomotor, throwing, kicking, and balance. Research staff set up a course in a school gymnasium where participants were asked to perform the following five tasks: (1) run to a cone approximately 5 m away, turn around, and run back to the starting point; (2) hop to the same cone on 1 leg and hop back to the starting point; (3) throw a tennis ball overhand to a wall 1.5 m away and have it bounce back over their head; (4) kick a ball to a wall 4 m away over a line 1 m from the ground; and (5) walk toe-to-heel in a straight line for 2 m. The tasks were assessed on a scale ranging from 1 to 100, with 0 to 25 representing *initial ranking*, 25 to 50 representing *emerging*, 50 to 75 representing *competent*, and 75 to 100 representing *proficient*. Final scores were calculated by adding section totals to obtain a total score and then dividing by 5, according to the scale’s instructions [21].

#### BMI Assessment

Height and weight were assessed objectively by trained research staff using a validated scale [22] and stadiometer [23]. BMIs were calculated with the following formula:

BMI = weight (kg)/height (m)^2^

#### Demographics

Age, race, ethnicity, and sex were assessed by using questions from the Youth Risk Behavior Surveillance System [1].

### Statistical Analysis

Univariate statistics were conducted for all study variables. Chi-square difference tests were conducted on demographic variables between groups. Mean difference scores for each outcome variable were calculated. A series of 2-tailed *t* tests were conducted to assess between-group differences. All analyses were conducted in SPSS (IBM Corp) [24]. An α level of 95% was used for all analyses. All self-report data were collected in Qualtrics (Qualtrics International Inc) [25]. Accelerometry data were collected from the Garmin application programming interface.

## Results

**Figure 1:**
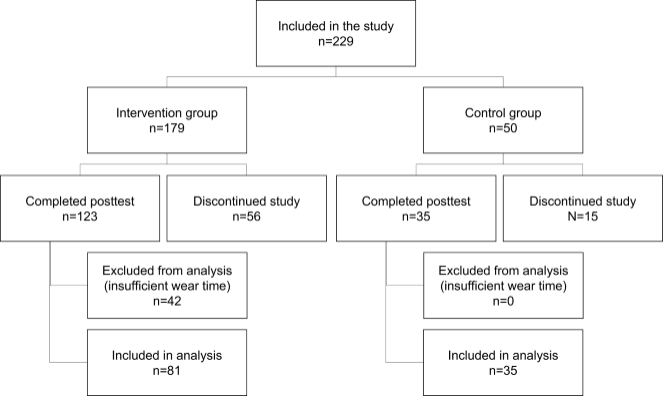
Flowchart of participants in this study.

Of the 179 intervention youths that initially consented to participating in this study, 42 were excluded, and 56 were lost to follow-up, resulting in 81 intervention youths being included in the analyses. Of the 50 control youths that initially consented to participating in this study, 15 were lost to follow-up, resulting in 35 control youths being included in the analyses (Figure 1).

Table 2 presents demographic information on the intervention and control participants. In the intervention group, participants were aged 13.4 (SD 1.0) years and distributed among the sixth (31/81, 38%), seventh (26/81, 32%), and eighth (24/81, 30%) grades. Further, 64.2% (52/81) reported being male, 76.5% (62/81) reported being African American or Black, 15% (12/81) reported being Hispanic or Latinx, and 19% (15/81) reported being White. In the control group, participants were aged 13.8 (SD 0.97) years, and more participants were in the eighth grade, with 11% (4/35), 31% (11/35), and 57% (20/35) in grades 6, 7, and 8, respectively. Moreover, 49% (17/35) reported being male, 51% (18/35) reported being African American or Black, 9% (3/35) reported being Hispanic or Latinx, and 11% (4/35) reported being White.

Chi-square difference tests were conducted for all categorial variables to understand if there was a difference in demographic variables between the intervention and control groups. There were no significant differences among demographic variables between groups.

Table 3 presents differences in BMIs, physical literacy, and accelerometry-measured physical activity between the intervention and control groups. The mean BMI was 23.37 (SD 5.91) kg/m^2^ for intervention participants and 25.19 (SD 7.10) kg/m^2^ for control participants. The small difference in BMIs between groups was not statistically significant (*t*_115_=1.41; *P*=.90).

Physical literacy was statistically different between the intervention and control groups (*t*_115_=7.57; *P*=.004). Participants in the intervention group had an average physical literacy score of 75.62 (SD 14.13), indicating a proficient ranking, while the participants in the control group had an average physical literacy score of 50.71 (SD 19.73), indicating a competent ranking. The mean difference in physical literacy between groups was 24.91 on the 100-point scale.

Minutes per week of MVPA (*t*_115_=4.28; *P*=.04) and steps per day (*t*_115_=4.29; *P*=.03) were statistically different between groups. On average, participants in the intervention group engaged in 107.01 (SD 34.94) minutes of MVPA per week and 10,847.11 (SD 3758.33) steps per day. Participants in the control group engaged in 53.01 (SD 11.17) minutes of MVPA per week and 5030.09 (SD 1128.24) steps per day. The mean differences between groups were 53.99 minutes of MVPA per week and 5817.01 steps per day.

**Table 2:** End point univariate statistics.

Characteristic			Intervention group (n=81)		Control group (n=35)		Chi-square (*df*)			*P* value	
Age (years), mean (SD)			13.4 (1.0)		13.8 (0.97)		7.61 (115)			.11	
**Grade in school, n (%)**								3.06 (115)			.22
	Sixth grade	31 (38)		4 (11)							
	Seventh grade	26 (32)		11 (31)							
	Eighth grade	24 (30)		20 (57)							
**Sex, n (%)**								1.95 (115)			.38
	Male	52 (64)		17 (49)							
	Female	28 (35)		18 (51)							
	Prefer not to say	1 (1.2)		0 (0)							
**Race^a^, n (%)**								15.63 (115)			.11
	African American or Black	62 (77)		18 (51)							
	Hispanic or Latinx	12 (15)		3 (9)							
	White, Non-Hispanic	15 (19)		4 (11)							
	Asian	5 (6)		1 (3)							
	Native Hawaiian or Other Pacific Islander	3 (4)		0 (0)							
	American Indian or Alaska Native	3 (4)		4 (11)							

^a^Participants were able to choose multiple racial categories.

**Table 3:** Differences between the intervention and control groups.

Outcome	Intervention group, mean (SD)	Control group, mean (SD)	Mean difference (95% CI)	*t* test (*df*)	*P* value
BMI (kg/m^2^)	23.37 (5.91)	25.19 (7.10)	1.82 (−4.60 to 0.96)	1.41 (115)	.90
Physical literacy score	75.62 (14.13)	50.71 (19.73)	24.91 (18.39 to 31.43)	7.57 (115)	.004
Moderate to vigorous physical activity (minutes per week)	107.01 (34.94)	53.01 (11.17)	53.99 (28.45 to 79.54)	4.28 (115)	.04
Steps per day	10,847.11 (3758.33)	5030.09 (1128.24)	5817.01 (3073.15 to 8560.88)	4.29 (115)	.03

## Discussion

The purpose of this study was to examine the potential effectiveness of an after-school sport sampling program on physical activity, physical literacy, and BMI among underserved racial and ethnic minority youth in the Midwest. Overall, intervention participants had significantly higher physical literacy scores (*P*=.004) and engaged in more MVPA (*P*=.04) and steps (*P*=.03) than youth in the control group after the intervention. This study aids in the understanding of physical activity for youth in a large, urban Midwest city and provides some evidence that a participant-informed, culturally tailored sport sampling intervention may be a mechanism for increasing physical activity and physical literacy among youth.

This study observed significant differences in MVPA between the intervention and control groups. This difference is consistent with past research that found that after-school programming is an effective strategy for increasing physical activity [26,27]. This study also adds important evidence on the use of sport sampling interventions to potentially reduce health inequality for Black and Hispanic youth.

*Move More, Get More* allowed youth to provide input on which sports they wanted to learn and practice. By incorporating youths’ preferences for which sports they want to engage in, we are potentially better able to maintain their interest in sports and physical activity. Future research should empirically examine if this participant-led approach results in maintained engagement in sports and physical activity for the long-term.

Physical literacy was significantly better in the intervention group compared to that in the control group (*P*=.004), indicating that a sport sampling intervention may be a good strategy for increasing physical literacy. Rajabiyan and Talebi [28] similarly found that an intervention of selected sports was effective in improving physical literacy. More broadly, a recent systematic review found that physical literacy–related interventions can be successful [29]. Evidence supports that physical literacy is associated with physical activity [17] and is believed to contribute to lifelong physical activity [16]. Future research should examine the long-term impacts of physical activity interventions on physical literacy and related health outcomes.

Unexpectedly, there was no significant difference in BMIs between youth in the intervention group and youth in the control group at posttest (*P*=.90). The Centers for Disease Control and Prevention recommend increasing BMI cutoff points for youth until the age of 20 years [30]. Although increases in BMI are usually indicative of poorer health behaviors, as middle school students grow and mature, increases in BMI may be an expected part of normal development. Future studies may consider alternative measures to assess body composition for this age group.

There are several strengths to this study. First, this study was conducted in an urban environment where 100% of the school districts’ students qualify for free lunches or lunches at reduced costs and schools serve predominantly racial and ethnic minority individuals. Second, this study adds to the limited research on the effectiveness of sport sampling interventions on physical literacy and physical activity. Additionally, this study assessed physical activity via accelerometry and objectively measured physical literacy and BMIs by using reliable and validated tools.

This study however relies on a posttest-only design that may contribute to type 2 error. A posttest-only design is one way to assess differences among groups while also delivering a needed intervention for underserved populations. Although not ideal, several physical activity studies have used a posttest-only design to increase the external reliability of real-world interventions [31-33].

Over 10 million youths participate annually in after-school programming [26]. These programs have the potential to provide a unique opportunity to help millions of youths become more active and improve MVPA. Although limited, our study and other evidence suggest that after-school sport sampling interventions are effective strategies for increasing physical literacy and physical activity among underserved racial and ethnic minority youth. Future research needs to be conducted on the best way to scale such interventions to board populations of youth to improve physical activity, physical literacy, and health equity.

## Abbreviations

KCPS: Kansas City Public School District
MVPA: moderate to vigorous physical activity
